# Approach to endoscopic extraperitoneal radical prostatectomy (EERPE): the impact of previous laparoscopic experience on the learning curve

**DOI:** 10.1186/1471-2490-7-11

**Published:** 2007-07-09

**Authors:** Andreas Blana, Markus Straub, Peter J Wild, Jens C Lunz, Thorsten Bach, Wolf F Wieland, Roman Ganzer

**Affiliations:** 1 Department of Urology, University of Regensburg, St Josef's Hospital, Landshuter Strasse 65, 93053 Regensburg, Germany; 2 Department of Pathology, University Hospital Zurich, Schmelzbergstrasse 12, 8091 Zurich, Switzerland; 3 Department of Urology, Asklepios Hospital Barmbek, Hamburg, Germany

## Abstract

**Background:**

We report our approach regarding the technique of endoscopic extraperitoneal radical prostatectomy (EERPE) and analyze the learning curve of two surgeons after thorough technical training under expert monitoring. The purpose of this study was to investigate the influence of expert monitoring on the surgical outcome and whether previous laparoscopic experience influences the surgeon's learning curve.

**Methods:**

EERPE was performed on 120 consecutive patients by two surgeons with different experience in laparoscopy. An analysis and comparison of their learning curve was made.

**Results:**

Median operation time: 200 (110 – 415) minutes. Complications: no conversion, blood transfusion (1.7%), rectal injury (3.3%). Median catheterisation time: 6 (5 – 45) days. Histopathological data: 55% pT2, 45% pT3 with a positive surgical margin rate of 6.1% and 46%, respectively. After 12 months, 78% of the patients were continent, 22% used 1 or more pad. Potency rate with or without PDE-5-inhibitors was 66% with bilateral and 31% with unilateral nerve-sparing, respectively. Operation time was the only parameter to differ significantly between the two surgeons.

**Conclusion:**

EERPE can be learned within a short teaching phase. Previous laparoscopic experience is reflected by shorter operation times, not by lower complication rates or superior early oncological data.

## Background

The initial step towards minimally invasive surgical treatment of localized prostate cancer was made by Schuessler et al. in the early Nineties with his description of a laparoscopic radical prostatectomy (LRP) [1]. However, the first larger series of LRP was published by Guillonneau et al. in 1999 [2]. After Raboy et al. described an extraperitoneal approach to the prostate in 1997 [3], Bollens et al. presented a series of 50 cases of EERPE in 2001 [4]. Based on his technique, further modifications were developed by Stolzenburg et al. [5-7] who established EERPE as a first – line minimally – invasive procedure for localized prostate cancer, suitable even for patients who had undergone previous abdominal surgery [8].

In view of the satisfactory oncological results of EERPE [9] and general post-operative advantages of laparoscopic compared to open surgery, we decided to establish EERPE at our hospital in March 2004. In this article we describe our experiences in learning EERPE and present the operative data and one year follow-up of the first 120 cases including a comparison of the learning curves of two surgeons with differing degrees of laparoscopic experience.

## Methods

### Surgeon characteristics and initial steps

Two surgeons commenced operative training simultaneously. Surgeon 1 (S1) had 7 years of laparoscopic experience, whereas surgeon 2 (S2) had only two years of experience in urological laparoscopy. Details of the laparoscopic procedures performed by both surgeons are given in Table 1. Both had performed over 50 cases of open retropubic radical prostatectomy, whereas neither of them was experienced in laparoscopic radical prostatectomy.

**Table 1:** Laparoscopic operations performed by surgeon 1 & surgeion 2 before starting training for EERPE

Laparoscopic operations	Number of operations	
	
	Surgeon 1	Surgeon 2
Varicocele ligation	82	19
Pelvic staging lymph node dissection	25	4
Modified retroperitoneal lymph node dissection	3	
Tumor nephrectomy	1	
Simple nephrectomy	12	
Nehproureterectomy	4	
Pyeloplasty	13	
Cyst decortication	5	
Adhesiolysis	13	
Lymphocele fenestration	19	2

After studying multi media material, both surgeons were trained in a dry lab (pelvic trainer) and on a porcine model for 4 weeks. During one week of training in a high-volume centre, each surgeon attended 6 procedures (camera and assistance) of EERPE. Back at our hospital, 9 consecutive procedures were supervised by experts in the technique. Patient pre – selection was not made for either surgeons.

### Patient characteristics

Between April 2004 and April 2005, 120 consecutive patients underwent EERPE performed by two surgeons. This study was carried out with the approval of the local ethics committee of the University of Regensburg and all patients gave written informed consent before participating in the study. Baseline characteristics are shown in Table 2. 47 (39.2%) patients had had previous surgery: open inguinal hernia repair (n = 21), open appendectomy (n = 17), transurethral resection of the prostate (n = 3), open cholecystectomy (n = 2), laparoscopic cholecystectomy (n = 1), gastrectomy (n = 1), umbilical hernia repair (n = 1) and partial bowel resection (n = 1). 10 (8.3%) patients had neo-adjuvant hormonal therapy (3 – 12 weeks).

**Table 2:** Patient characteristics and baseline data

number of patients	120
median age in years (range)	65 (4.1 – 76)
median PSA in ng/mL (range)	8.68 (1.1 – 29.9)
median prostate volume in mL (range)	30.9 (9 – 87)
median Gleason score (range)	6 (3 – 10)

### Oncological and functional follow-up

All patients were followed up every three months by a self-administered questionnaire sent by mail, including a stamped return envelope addressed to our institution. Erectile function was evaluated by the short form of the international index of erectile function questionnaire (IIEF 5). The possible scores for the IIEF-5 range from 5 to 25, and erectile dysfunction (ED) was classified into five categories based on the following scores: severe (5–7), moderate (8–11), mild to moderate (12–16), mild (17–21) and no ED (22–25) [10]. Continence was evaluated by a question concerning pad use. Patients were considered continent if they did not require protection. Our follow-up schedule included PSA measurements at 3-monthly intervals and questions on adjuvant therapy. Biochemical failure was defined as any measurable PSA greater than 0.2 ng/mL.

### Technique

EERPE was performed using the technique described by Stolzenburg et al. Pelvic lymph node dissection was performed in patients with PSA ≥ 10 ng/ml and/or a Gleasonscore ≥ 7. Criteria for a nerve-sparing technique were as follows: no preoperative erectile dysfunction, PSA ≤ 10 ng/mL, Gleason score ≤ 6 and no palpable tumour on the nerve-sparing side.

### Statistical analysis of the learning curve

After analyzing all important parameters a separate analysis of the learning curve was made according to the specification of three phases. Phase 1: Expert supervision of the first 9 operations (S1 n = 7, S2 n = 2). Phase 2: 16 operations performed by the same team with S1 and S2 assisting each other (S1 n = 9, S2 n = 7). Phase 3: 95 operations with varying assistants (S1 n = 50, S2 n = 45). Thereafter the data of S1 and S2 were compared. Contingency table analysis and two-sided Fisher's exact tests were used to study the association between categorical variables. The two-sided Mann-Whitney U-test was used for the non-parametric comparison of two independent metric variables. Differences between three independent metric variables were tested with the two-sided Kruskal-Wallis test. P values < 0.05 were considered significant. Statistical analyses were completed using SPSS version 10.0 (SPSS, Chicago, IL, USA). The closed test principle was used for multiple testing.

## Results

### Peri-operative data

The median operation time was 200 minutes (range 110 – 415). Pelvic lymph node dissection was performed in 26 (21.7%) patients, unilateral nerve-sparing in 26 (21.7%) and bilateral nerve-sparing in 5 (4.2%) cases. The median aspirated blood/urine mixture was 300 ml (range 100 – 3000). 2 (1.7%) patients received blood transfusion within 24 h post-operatively. There was no conversion to open surgery or early reintervention.

### Postoperative management

The median catheterisation time was 6 days (range 5 – 45, mean 7.1). A routinely performed radiological control showed a leakage of the vesicourethral anastomosis in 9 (7.5%) patients on day 6.

### Intra-operative complications

A patient with a history of stable coronary heart disease and previous myocardial infarction developed intraoperative cardiac shock due to myocardial reinfarction and died 14 h postoperatively. During the development of the preperitoneal space major bleeding occurred in one patient due to injury of the epigastric vessels, this being stopped immediately by endoclips. Intra-operative rectal injury occurred in 4 (3.3%) patients during the preparation of the prostate apex. Once recognized the defect was closed in two layers (Vicryl 2/0). A rectal catheter was inserted and intravenous antibiotics were administered for 3 days. Food intake was not delayed. No patient required a diverting colostomy.

### Early post-operative complications

After intra-operative deterioration of the respiratory parameters, one patient developed acute respiratory distress syndrome (ARDS) but recovered completely after prolonged intensive-care treatment. Three weeks after regular discharge from hospital one patient presented with a vesicorectal fistula requiring temporary colostomy and suprapubic catheterisation of the bladder for 9 weeks with consecutive spontaneous closure of the defect. Symptomatic pelvic lymphocele occurred in 2 (1.7%) and was treated by laparoscopic intervention. Due to 2^nd ^degree hydronephrosis one patient required a temporary nephrostomy. No patient developed ileus, deep venal thrombosis, pulmonary embolism or nosocomial pneumonia.

### Histopathological results

Histopathological stage was defined according to the TNM classification (UICC 2002). The distribution is shown in Table 3.

**Table 3:** Oncological results

Tumour stage	
**pT2 (%)**	**66 (55)**
pT2a (%)	21 (17.5)
pT2b (%)	3 (2.5)
pT2c (%)	42 (35)
**pT3 (%)**	**54 (45)**
pT3a (%)	39 (32.5)
pT3b (%)	15 (12.5)

Lymph node status	

PNx (%)	94 (78.3)
pN0 (%)	22 (18.3)
pN 1–2 (%)	4 (3.3)

Gleason score	

<7 (%)	70 (58.3)
≥7 (%)	50 (41.7)

Surgical margin status	

R0 (%)	91 (75.8)
R1 (%)	29 (24.2)
**R1 among pT2 (%)**	**4 (6.1)**
R1 among pT2a (%)	1 (4.7)
R1 among pT2b (%)	0
R1 among pT2c (%)	3 (7.1)
**R1 among pT3 (%)**	**25 (46)**
R1 among pT3a (%)	20 (51)
R1 among pT3b (%)	5 (33.3)

### Oncological follow-up

In our series, 7 (5.8%) patients experienced PSA failure during the first year. In 4 of these patients, adjuvant therapy was initiated (anti-hormonal therapy in 3 patients, radiation in 1 patient).

### Functional follow – up

84 (70%) patients completed the functional follow-up. Of these patients, 62 (74%) patients were completely continent (no pads) 6 months after surgery. 17 (20%) patients reported the daily use of 1 to 2 pads, whereas 5 (6%) patients used more than 2 pads. 12 months post-operatively, 64 (78%) patients were continent, 14 (17%) used 1 to 2 pads and 4 (5%) patients used more than 2 pads.

A 12-month functional follow-up was completed by 3 of 5 (60%) patients with bilateral nerve-sparing and by 13 of 26 (50%) patients with unilateral nerve-sparing. In the bilateral nerve-sparing group, 2 of 3 (66%) had an IIEF score > 17 and sexual intercourse was possible with or without PDE 5 inhibitors. 1 of 13 (8%) patients with unilateral nerve-sparing had an IIEF score > 17 and 4 of 13 (31%) reported erectile function sufficient for sexual intercourse with the help of PDE 5 inhibitors.

### Analysis of the learning curve

#### Comparison of 3 phases

**Figure 1:**
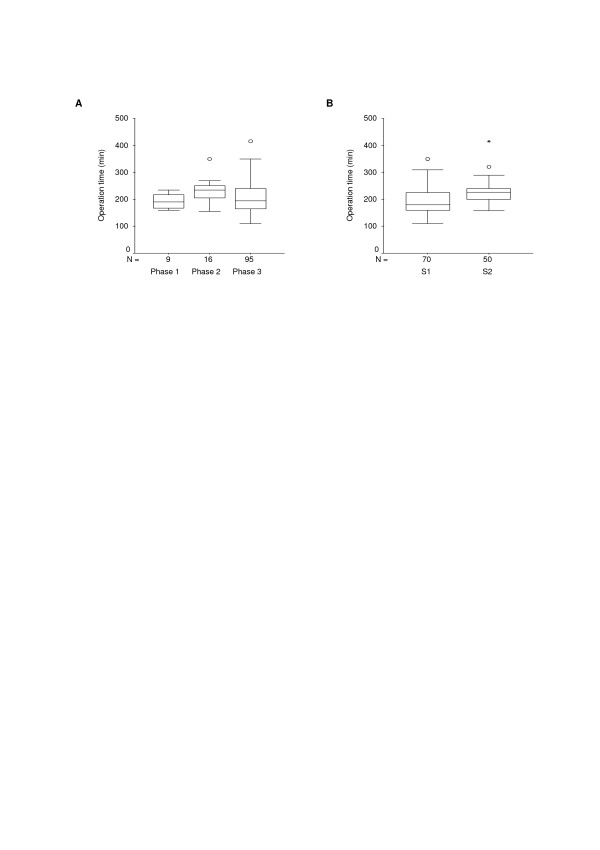
1A and 1B. Boxplots for operation time of learning phases and surgeons. KEY: S1 = Surgeon 1; S2 = Surgeon 2.

Analysis of the median operation time shows an initial increase from 190 (range 160 – 235) minutes in phase 1 (9 operations under expert supervision) to 234 (range 155 – 350) minutes in phase 2 (16 operations with S1 and S2 assisting each other). Thereafter, median operation time decreased slightly to 195 (range 110 – 415) minutes in phase 3 (95 operations with varying assistants, Figure 1A). However, the differences were not statistically significant (p = 0.054; Table 4). Furthermore, the rate of positive surgical margins was not significantly different in relation to the different training phases (p = 1.000; Table 4). The number of intra-operative and postoperative complications within the 3 training phases did not differ significantly (p = 0.081, p = 1.000; Table 4).

**Table 4:** Comparison of phases and surgeons

	**Phase 1**	**Phase 2**	**Phase 3**	**p**	**S1**	**S2**	**p**
no. of operations	9	16	95		70	50	
median operation time in minutes (range)	190 (160–235)	234 (155–350)	195 (110–415)	0.054	180 (110–350)	227 (158–415)	**< 0.001**
median catheterisation time in days (range)	6 (6-6)	6 (6-6)	6 (6–45)	0.066	6.0 (6–45)	6.0 (6–40)	0.285
no. of intraoperative complications	0	0	6	0.746	1	5	0.081
no. of postoperative complications	1	0	7	0.372	5	3	1.000
no. of positive surgical margins (%)	2 (22.2)	3 (20)	24 (25.3)	0.924	16 (22.8)	13 (26)	1.000

bold face representing significant data.

#### Comparison of both surgeons

The overall median operation time was significantly shorter for S1 (p < 0.001, Table 4, Figure 1A). Apart from the operation time, no significant difference concerning intra- and post-operative complications could be found between either of the two surgeons (Table 4). In detail, intra-operative injury of the epigastric vessels was caused by S1, all rectal injuries (4) by S2. Cardiac shock occurred in a patient operated on by S1. Reintervention for recto-vesical fistula and hydronephrosis was necessary after operations performed by S1, whereas the occurrence rate of symptomatic pelvic lymphocele was equal for both surgeons. The positive surgical margin rate was not significantly different between either of the two surgeons (p = 1.000).

## Discussion

Laparoscopic radical prostatectomy has made impressive progress in the treatment of organ-confined prostate cancer since the first description by Schuessler et al. in 1991 [11]. The main objective in establishing the technique was to combine cure rates of open radical prostatectomy with the low morbidity of minimally-invasive procedures. Meanwhile, laparoscopic radical prostatectomy is the first-line option in the treatment of organ-confined prostate cancer in selected centres [12-14]. Nevertheless, despite being attractive, EERPE is restricted to a few centres as a result of its demanding technical requirements and the length of the learning curve. Therefore, a crucial issue when establishing the procedure in a hospital is the initial approach and overcoming of the learning curve. With the present study we are contributing towards to an assessment of the feasibility of this surgical approach. The special feature in our learning curve is the short teaching phase of only 9 operations under expert monitoring.

### Operation time

Using the transperitoneal approach, Guillonneau et al. report a mean operation time of 203 minutes after 567 cases [15], with 268 minutes in the first 50 operations. Rassweiler et al. report 324 minutes of mean operation time in the first 60 sessions and 265 minutes in the last 120 [16]. In the case of the extraperitoneal approach significantly shorter mean operation times are reported by Stolzenburg et al., namely 151 minutes after 700 cases. With a mean operation time of 206 minutes, our results are encouraging, whilst a further decrease in operation time is anticipated.

### Transfusion and conversion rate

Most groups report a decrease in blood transfusion rates during the learning curve[17,18]. In relevant literature, transfusion rates from 0.9% (Stolzenburg et al.) [19] to 31% (Rassweiler et al.) [20] are reported. With a perioperative transfusion rate of 1.7% we achieved encouraging results in our series. Furthermore, similar to the series described by Turk et al. [21], Gregori et al. [22] and Stolzenburg et al. [23], we did not experience any immediate conversion to open surgery, whereas other groups report conversion rates up to 4.4% [24].

### Complications

Complications commonly occur during the first cases and decrease with the surgeon's experience [25]. In our series, this particular apect cannot be confirmed, considering our 4 (3.3%) cases of rectal injury which occurred not during the first operations, but in phase 3. However, this complication rate is still within the limits of other groups' experiences, which range from 0.6% [26] to 6.8% [27]. Beyond that, our series shows a generally low rate of intraoperative and early post-operative complications (Table 4).

### Histopathological results

When introducing laparoscopic radical prostatectomy a major goal must be to achieve oncological results comparable to those of other high volume centres. In this context, an important feature is the rate of positive surgical margins derived from pathological examination of the surgical specimen, as it represents an important prognostic factor [28]. Positive surgical margins after LRP range from 11.4% (Dahl et al.) [29], 20% (Stolzenburg et al.) [30] up to 34% by a "junior surgeon" under supervision during his early learning curve (El-Feel et al.) [31]. Our overall positive surgical margin rate was 24.2% with a relatively low rate of 6.1% concerning stage pT2 tumours.

### Functional und oncological follow-up

Classification of the degree of incontinence after radical prostatectomy is an important factor for quality control. However, there is no widespread use of a validated continence questionnaire and in almost all publications the continence status is evaluated by the number of pads used by the patient [32-34]. In order to compare our data with other groups, we evaluated the "pad situation" in our series, which is comparable with other publications [35]. However, the number of pads is not an unequivocable tool to measure incontinence, as it depends strongly on the patient's habits and the amount of urine in the pad when it is changed.

Regarding PSA follow-up, it is essential to consider, that one year is a relatively short follow-up time to evaluate the oncological aspect of radical prostatectomy, as biochemical failure during the first year is mostly due to systemic and not to local recurrence [36]. However, our biochemical recurrence rate of 5.8% resembles the experiences of other groups [37] and reflects the high number of pT3 tumors in this series. However, Stolzenburg et al., pioneers in EERPE, had the same rate in pT3 tumours in their series of 700 patients [38].

### Learning curve

Our initial steps towards gaining proficiency in EERPE are similar to those described by Bollens et al. with respect to the steps of dry lab, animal live lab and mentoring with an expert [39]. Our teaching phase commenced with relatively short median operation times (190 minutes, phase 1), showing the influence of expert supervision. The rise in median operation time to 234 minutes (phase 2) reflects a phase of uncertainty and lack of supervision. However, after only 25 operations we felt comfortable enough to rotate the operating teams in order to instruct new assistants (phase 3) and achieved shorter operation times. Mean catheterisation time and the occurrence of complications were not influenced by the learning curve as was the case in other groups [40]. Furthermore, the rate of positive surgical margins remained stable and did not differ significantly in the different stages of the learning curve.

Comparing the learning curves of both surgeons, the impact of superior experience in laparoscopy is only reflected by significantly shorter operation times. Despite differing degrees of laparoscopic experience, the two surgeons did not differ significantly as regards positive surgical margin rates, this, in contrast, being described by other groups [41].

A shortcoming of our study is the retrospective character of the analysis with a relatively small number of patients. Although the results of this paper reflect a typical approach to establish laparoscopic procedures in a hospital, prospective studies are needed to analyse learning curves of surgeons and to find a way to overcome these.

## Conclusion

Our series of EERPE performed on 120 patients shows results comparable to those of other high-volume centres as regards peri-operative data, patient morbidity and early functional and oncological outcomes. EERPE can be learned after thorough technical training under expert monitoring within a short teaching phase. Previous laparoscopic experience is reflected by shorter operation times but similar complication rates and oncological results.

## Competing interests

The author(s) declare that they have no competing interests.

## Authors' contributions

All authors contributed equally to this work and read and approved the final manuscript

## Pre-publication history

The pre-publication history for this paper can be accessed here:



## References

[B1] Schuessler WW, Schulam PG, Clayman RV, Kavoussi LR (1997). Laparoscopic radical prostatectomy: initial short-term experience. Urology.

[B2] Guillonneau B, Cathelineau X, Barret E, Rozet F, Vallancien G (1999). Laparoscopic radical prostatectomy: technical and early oncological assessment of 40 operations. Eur Urol.

[B3] Raboy A, Ferzli G, Albert P (1997). Initial experience with extraperitoneal endoscopic radical retropubic prostatectomy. Urology.

[B4] Bollens R, Vanden BM, Roumeguere T, Damoun A, Ekane S, Hoffmann P, Zlotta AR, Schulman CC (2001). Extraperitoneal laparoscopic radical prostatectomy. Results after 50 cases. Eur Urol.

[B5] Stolzenburg JU, Rabenalt R, Tannapfel A, Liatsikos EN (2006). Intrafascial nerve-sparing endoscopic extraperitoneal radical prostatectomy. Urology.

[B6] Stolzenburg JU, Do M, Pfeiffer H, Konig F, Aedtner B, Dorschner W (2002). The endoscopic extraperitoneal radical prostatectomy (EERPE): technique and initial experience. World J Urol.

[B7] Stolzenburg JU, Rabenalt R, Do M, Ho K, Dorschner W, Waldkirch E, Jonas U, Schutz A, Horn L, Truss MC (2005). Endoscopic extraperitoneal radical prostatectomy: oncological and functional results after 700 procedures. J Urol.

[B8] Stolzenburg JU, Ho KM, Do M, Rabenalt R, Dorschner W, Truss MC (2005). Impact of previous surgery on endoscopic extraperitoneal radical prostatectomy. Urology.

[B9] Stolzenburg JU, Do M, Pfeiffer H, Konig F, Aedtner B, Dorschner W (2002). The endoscopic extraperitoneal radical prostatectomy (EERPE): technique and initial experience. World J Urol.

[B10] Rosen RC, Riley A, Wagner G, Osterloh IH, Kirkpatrick J, Mishra A (1997). The international index of erectile function (IIEF): a multidimensional scale for assessment of erectile dysfunction. Urology.

[B11] Schuessler WW, Schulam PG, Clayman RV, Kavoussi LR (1997). Laparoscopic radical prostatectomy: initial short-term experience. Urology.

[B12] Guillonneau B, Gupta R, El FH, Cathelineau X, Baumert H, Vallancien G (2003). Laparoscopic [correction of laproscopic] management of rectal injury during laparoscopic [correction of laproscopic] radical prostatectomy. J Urol.

[B13] Rassweiler J, Schulze M, Teber D, Marrero R, Seemann O, Rumpelt J, Frede T (2005). Laparoscopic radical prostatectomy with the Heilbronn technique: oncological results in the first 500 patients. J Urol.

[B14] Stolzenburg JU, Rabenalt R, Do M, Ho K, Dorschner W, Waldkirch E, Jonas U, Schutz A, Horn L, Truss MC (2005). Endoscopic extraperitoneal radical prostatectomy: oncological and functional results after 700 procedures. J Urol.

[B15] Guillonneau B, Rozet F, Cathelineau X, Lay F, Barret E, Doublet JD, Baumert H, Vallancien G (2002). Perioperative complications of laparoscopic radical prostatectomy: the Montsouris 3-year experience. J Urol.

[B16] Rassweiler J, Sentker L, Seemann O, Hatzinger M, Rumpelt HJ (2001). Laparoscopic radical prostatectomy with the Heilbronn technique: an analysis of the first 180 cases. J Urol.

[B17] Bollens R, Vanden BM, Roumeguere T, Damoun A, Ekane S, Hoffmann P, Zlotta AR, Schulman CC (2001). Extraperitoneal laparoscopic radical prostatectomy. Results after 50 cases. Eur Urol.

[B18] Eden CG, Cahill D, Vass JA, Adams TH, Dauleh MI (2002). Laparoscopic radical prostatectomy: the initial UK series. BJU Int.

[B19] Stolzenburg JU, Rabenalt R, Do M, Ho K, Dorschner W, Waldkirch E, Jonas U, Schutz A, Horn L, Truss MC (2005). Endoscopic extraperitoneal radical prostatectomy: oncological and functional results after 700 procedures. J Urol.

[B20] Rassweiler J, Sentker L, Seemann O, Hatzinger M, Rumpelt HJ (2001). Laparoscopic radical prostatectomy with the Heilbronn technique: an analysis of the first 180 cases. J Urol.

[B21] Turk I, Deger S, Winkelmann B, Schonberger B, Loening SA (2001). Laparoscopic radical prostatectomy. Technical aspects and experience with 125 cases. Eur Urol.

[B22] Gregori A, Simonato A, Lissiani A, Bozzola A, Galli S, Gaboardi F (2003). Laparoscopic radical prostatectomy: perioperative complications in an initial and consecutive series of 80 cases. Eur Urol.

[B23] Stolzenburg JU, Rabenalt R, Do M, Ho K, Dorschner W, Waldkirch E, Jonas U, Schutz A, Horn L, Truss MC (2005). Endoscopic extraperitoneal radical prostatectomy: oncological and functional results after 700 procedures. J Urol.

[B24] Rassweiler J, Sentker L, Seemann O, Hatzinger M, Rumpelt HJ (2001). Laparoscopic radical prostatectomy with the Heilbronn technique: an analysis of the first 180 cases. J Urol.

[B25] Guillonneau B, Rozet F, Cathelineau X, Lay F, Barret E, Doublet JD, Baumert H, Vallancien G (2002). Perioperative complications of laparoscopic radical prostatectomy: the Montsouris 3-year experience. J Urol.

[B26] Stolzenburg JU, Rabenalt R, Do M, Ho K, Dorschner W, Waldkirch E, Jonas U, Schutz A, Horn L, Truss MC (2005). Endoscopic extraperitoneal radical prostatectomy: oncological and functional results after 700 procedures. J Urol.

[B27] Arai Y, Egawa S, Terachi T, Suzuki K, Gotoh M, Kawakita M, Tanaka M, Terada N, Baba S, Okumura K (2003). Morbidity of laparoscopic radical prostatectomy: summary of early multi-institutional experience in Japan. Int J Urol.

[B28] Sofer M, Hamilton-Nelson KL, Civantos F, Soloway MS (2002). Positive surgical margins after radical retropubic prostatectomy: the influence of site and number on progression. J Urol.

[B29] Dahl DM, L'esperance JO, Trainer AF, Jiang Z, Gallagher K, Litwin DE, Blute RD (2002). Laparoscopic radical prostatectomy: initial 70 cases at a U.S. university medical center. Urology.

[B30] Stolzenburg JU, Rabenalt R, Do M, Ho K, Dorschner W, Waldkirch E, Jonas U, Schutz A, Horn L, Truss MC (2005). Endoscopic extraperitoneal radical prostatectomy: oncological and functional results after 700 procedures. J Urol.

[B31] El-Feel A, Davis JW, Deger S, Roigas J, Wille AH, Schnorr D, Hakiem AA, Loening S, Tuerk IA (2003). Positive margins after laparoscopic radical prostatectomy: a prospective study of 100 cases performed by 4 different surgeons. Eur Urol.

[B32] Rozet F, Arroyo C, Cathelineau X, Barret E, Prapotnich D, Vallancien G (2004). Extraperitoneal standard laparoscopic radical prostatectomy. J Endourol.

[B33] Rozet F, Arroyo C, Cathelineau X, Barret E, Prapotnich D, Vallancien G (2004). Extraperitoneal standard laparoscopic radical prostatectomy. J Endourol.

[B34] Stolzenburg JU, Rabenalt R, Do M, Ho K, Dorschner W, Waldkirch E, Jonas U, Schutz A, Horn L, Truss MC (2005). Endoscopic extraperitoneal radical prostatectomy: oncological and functional results after 700 procedures. J Urol.

[B35] Stolzenburg JU, Rabenalt R, Do M, Ho K, Dorschner W, Waldkirch E, Jonas U, Schutz A, Horn L, Truss MC (2005). Endoscopic extraperitoneal radical prostatectomy: oncological and functional results after 700 procedures. J Urol.

[B36] Pound CR, Partin AW, Eisenberger MA, Chan DW, Pearson JD, Walsh PC (1999). Natural history of progression after PSA elevation following radical prostatectomy. JAMA.

[B37] Rozet F, Arroyo C, Cathelineau X, Barret E, Prapotnich D, Vallancien G (2004). Extraperitoneal standard laparoscopic radical prostatectomy. J Endourol.

[B38] Stolzenburg JU, Rabenalt R, Do M, Ho K, Dorschner W, Waldkirch E, Jonas U, Schutz A, Horn L, Truss MC (2005). Endoscopic extraperitoneal radical prostatectomy: oncological and functional results after 700 procedures. J Urol.

[B39] Bollens R, Sandhu S, Roumeguere T, Quackels T, Schulman C (2005). Laparoscopic radical prostatectomy: the learning curve. Curr Opin Urol.

[B40] Rassweiler J, Sentker L, Seemann O, Hatzinger M, Rumpelt HJ (2001). Laparoscopic radical prostatectomy with the Heilbronn technique: an analysis of the first 180 cases. J Urol.

[B41] El-Feel A, Davis JW, Deger S, Roigas J, Wille AH, Schnorr D, Hakiem AA, Loening S, Tuerk IA (2003). Positive margins after laparoscopic radical prostatectomy: a prospective study of 100 cases performed by 4 different surgeons. Eur Urol.

